# Treatment of Acromioclavicular Joint Instability With a Tunnel-Free Reconstruction Technique

**DOI:** 10.1016/j.eats.2022.01.008

**Published:** 2022-04-25

**Authors:** Michelle M. Gosselin, Brandon S. Denney, James M. Gregory

**Affiliations:** a Department of Orthopaedic Surgery, University of Texas Health Science Center at Houston, Houston, Texas, USA; b McGovern Medical School, University of Texas Health Science Center at Houston, Houston, Texas, USA

## Abstract

Acromioclavicular joint injuries are a common shoulder injury encountered by orthopaedic surgeons. Many different surgical techniques have been described for the operative treatment of these injuries with no single, clear gold standard technique on which surgeons agree. Among the most common complications after surgical management of acromioclavicular injuries are loss of reduction, infection, fracture of clavicle or coracoid, and need for reoperation. We propose an arthroscopic-assisted, tunnel-free surgical technique using a tibialis anterior allograft combined with a FiberTape Cerclage (Arthrex, Naples, FL) to manage both acute and chronic acromioclavicular joint injuries. No bony tunnels are drilled and no hardware is implanted, which should obviate the risk for subsequent bony failure through a fracture, nor require subsequent hardware removal. In addition, the combination of suspensory and allograft fixation should impart sufficient stability to maintain an adequate reduction even in the face of failure of one of the fixation methods.

Acromioclavicular joint injuries are a common shoulder injury managed by both general and specialty-trained orthopaedic surgeons.^1^^,^^2^ It is largely accepted that Rockwood grade I and II injuries should be treated nonoperatively; however, controversy remains over management of higher-grade acromioclavicular joint injuries, despite much research into this topic.^3^ More than 60 different surgical techniques have been described in the operative treatment of these injuries, the majority of which can be grouped into primary acromioclavicular and coracoclavicular open fixation, Weaver–Dunn/Modified Weaver–Dunn, anatomic reconstructions, suspensory fixation, and arthroscopic-assisted techniques.^1^, ^2^, ^3^, ^4^, ^5^, ^6^, ^7^, ^8^ Within each of these groups, fixation options include pins, screws, hook plates, suture buttons, suture loops, and suture anchors; graft options include allografts, autografts, and synthetics.^3^, ^4^, ^5^, ^6^, ^7^, ^8^ Each technique carries different advantages, disadvantages, and potential complications. Among the most common complications are loss of reduction, infection, fracture of clavicle or coracoid, and need for reoperation (planned or unplanned).^3^, ^4^, ^5^, ^6^, ^7^, ^8^

We propose an arthroscopic-assisted, tunnel-free surgical technique in effort to obviate the risk for subsequent bony failure through a fracture while maintaining an adequate reduction with a combination of suspensory and allograft fixation. This technique can be used in both acute and chronic injuries and avoids many common complications seen after surgical management of acromioclavicular injuries.

## Surgical Technique (With Video Illustration)

The overall steps of the procedure are summarized and presented in Table 1. A thorough description of each step is presented herein and illustrated in the accompanying video (Video 1). Pearls and pitfalls of the technique are presented in Table 2.

**Table 1:** Key Steps of the Procedure

**1**	Diagnostic arthroscopy is performed. The base of the coracoid is identified and debrided arthroscopically.
**2**	An incision is made over the distal clavicle extending to the AC joint. Blunt dissection is carried down below the anterior clavicle to the medial and lateral aspects of the coracoid. Bleeding bone beds are created on the coracoid and the anterior and posterior edges of the clavicle to promote healing.
**3**	The FiberTape cerclage and the looped end of a folded #5 FiberWire suture are passed around the coracoid from lateral to medial. The tails of the FiberWire suture are passed through the looped end to create a luggage tag around the coracoid.
**4**	The medial end of the cerclage suture and 1 of the 2 FiberWire tails are shuttled under the clavicle from anterior to posterior.
**5**	A tibialis anterior allograft is whip-stitched at both ends. The graft is then shuttled from lateral to medial beneath the base of the coracoid. The medial side of the graft is then shuttled beneath the clavicle from anterior to posterior.
**6**	The AC joint is held in reduction with a ball-spike pusher while the FiberTape cerclage is assembled and tensioned.
**7**	The 2 tails from the FiberWire luggage tag are tied over the top of the clavicle. The allograft is then tied and sewn in place over the top of the clavicle.
**8**	One tail of the allograft is cut at the level of the knot on top of the clavicle. The other end of the allograft is brought across the top of the clavicle to the AC joint. The graft is cut to appropriate length and sewn in to the posterior-superior aspect of the AC joint capsule.

AC, acromioclavicular.

**Table 2:** Pearls and Pitfalls

Pearls	Pitfalls
Thorough debridement of soft tissue from undersurface and base of coracoid	Insufficient reduction achieved with FiberTape Cerclage
Sufficient exposure to see posterior aspect of clavicle to ensure clear path underneath the clavicle from posterior to anterior	Inadequate incision to fully visualize acromioclavicular joint and associated anatomy
Confirm that the allograft rests superficial to the FiberWire and cerclage sutures with respect to the coracoid	
Confirm reduction visually and fluoroscopically before securing FiberTape Cerclage	

### Positioning and Setup

The patient is positioned in a traditional beach-chair position. The operative arm can be set up in a limb-positioning device per surgeon preference. Sterile preparation and draping of the shoulder should be appropriate to allow for standard arthroscopy portals and access to the coracoid, clavicle, and acromioclavicular joint. Specialized equipment and implants used for this case include 1 fresh-frozen tibialis anterior allograft, FiberTape Cerclage (Arthrex, Naples, FL) with accompanying suture passer, tensioning device and tensioner handle, #5 FiberWire (Arthrex), and #2 FiberLoop sutures (Arthrex). Standard arthroscopy equipment per surgeon preference is also necessary.

### Step 1: Diagnostic Arthroscopy and Coracoid Preparation

Shoulder arthroscopy is performed using a traditional posterior viewing portal. An anterior working portal is established with a cannula placed through the rotator interval; our preference is to use 5.75-mm × 7-cm Crystal Cannula (Arthrex). Any concomitant glenohumeral pathology can be identified and addressed as necessary. Attention is then turned to the medial aspect of the rotator interval, where a radiofrequency device is used to remove soft tissue from off of the base of the coracoid until the inferior and lateral edge of the coracoid base are adequately exposed. An arthroscopic shaver is then used to gently decorticated the undersurface of the coracoid base to promote healing of the subsequent coracoclavicular ligament reconstruction, as shown in the accompanying video (Video 1).

### Step 2: Open Exposure of the Acromioclavicular Joint, Clavicle, and Base of the Coracoid

**Fig 1:**
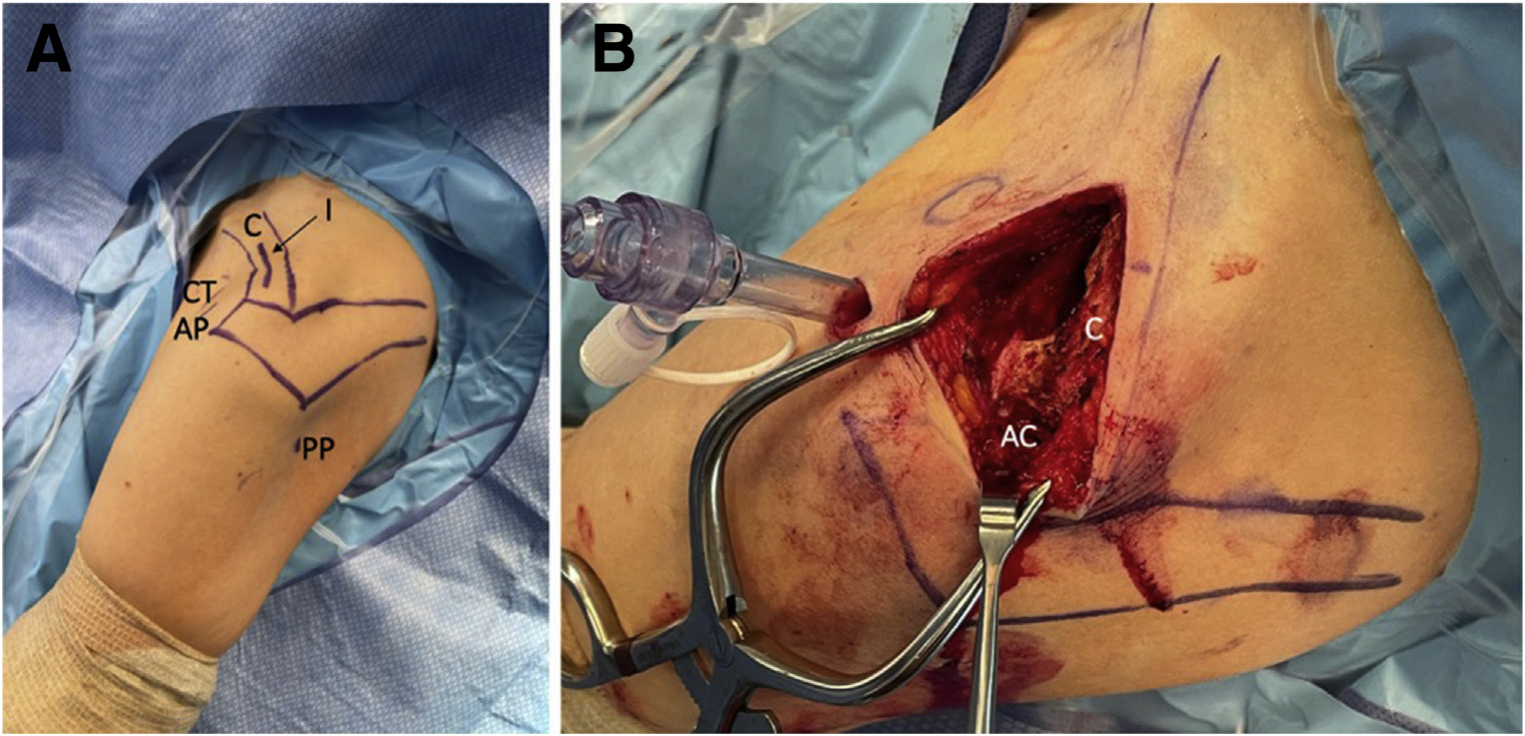
(A) A left shoulder is shown in the beach chair position. Shoulder landmarks have been marked including the clavicle (C), the tip of the coracoid (CT), the AC joint (AC), the anterior arthroscopic portal (AP), the posterior viewing portal (PP), and the planned incision (I) over the clavicle. (B) The same left shoulder is shown with the clavicle (C) and AC joint (AC) exposed through an incision down the longitudinal axis of the clavicle. Blunt dissection has been carried out just off the anterior edge of the clavicle down to the base of the coracoid (not pictured). Anterior and posterior arthroscopy portals also have been established.

An incision is planned over the long-axis of the distal clavicle, as illustrated in Figure 1A. The length of the incision should be determined based on patient anatomy to extend from the acromioclavicular joint to just medial to the coracoid, shown in Figure 1B. The incision is made with a 15 blade and sharp dissection is carried down to the superior aspect of the clavicle. The acromioclavicular joint capsule is then sharply incised in an anterior to posterior orientation. In the case of chronic acromioclavicular joint injuries, fibrous tissue may be encountered, which can impede reduction; this tissue, if present, should be sharply debrided. Careful dissection is then carried down to the anterior edge of the clavicle until the base of the coracoid can be identified. The posterior edge of the clavicle is also exposed at this level, ensuring a clear path underneath the clavicle from posterior to anterior. We prefer to use curved Mayo scissors for this step. An osteotome is then used to gently create a bleeding bone bed on the anterior and posterior edges of the clavicle and the superior aspect of the coracoid base.

### Step 3: Passage of FiberTape Cerclage and #5 FiberWire Around the Base of the Coracoid

Once the base of the coracoid has been identified through open dissection, the arthroscope can be reinserted into the glenohumeral joint through the posterior viewing portal. While viewing with the arthroscope, the curved Mayo scissors can be used to ensure that an appropriate dissection path has been created from the anterior aspect of the clavicle down to both the medial and lateral aspects of the coracoid base. A medium passing hook (AR-7806, Arthrex) loaded with a nitinol wire shuttle loop is then passed around the base of the coracoid from medial to lateral until the passing hook can be seen with the arthroscope just below the coracoid. The shuttle loop is then deployed and under arthroscopic visualization; an arthroscopic grasper can be used to retrieve the loop through the open incision from the lateral side of the coracoid base, as illustrated in the accompanying video summary (Video 1). A #5 FiberWire is then folded at its midpoint and the looped end of the FiberWire is then placed through the nitinol shuttle loop along with the free tail of the FiberTape Cerclage. The shuttle loop is then pulled to draw the FiberTape Cerclage and looped portion of the FiberWire from lateral to medial around the base of the coracoid, as shown in the accompanying video (Video 1).

### Step 4: Luggage Tag Creation Around the Coracoid and Shuttling Sutures Under the Clavicle

**Fig 2:**
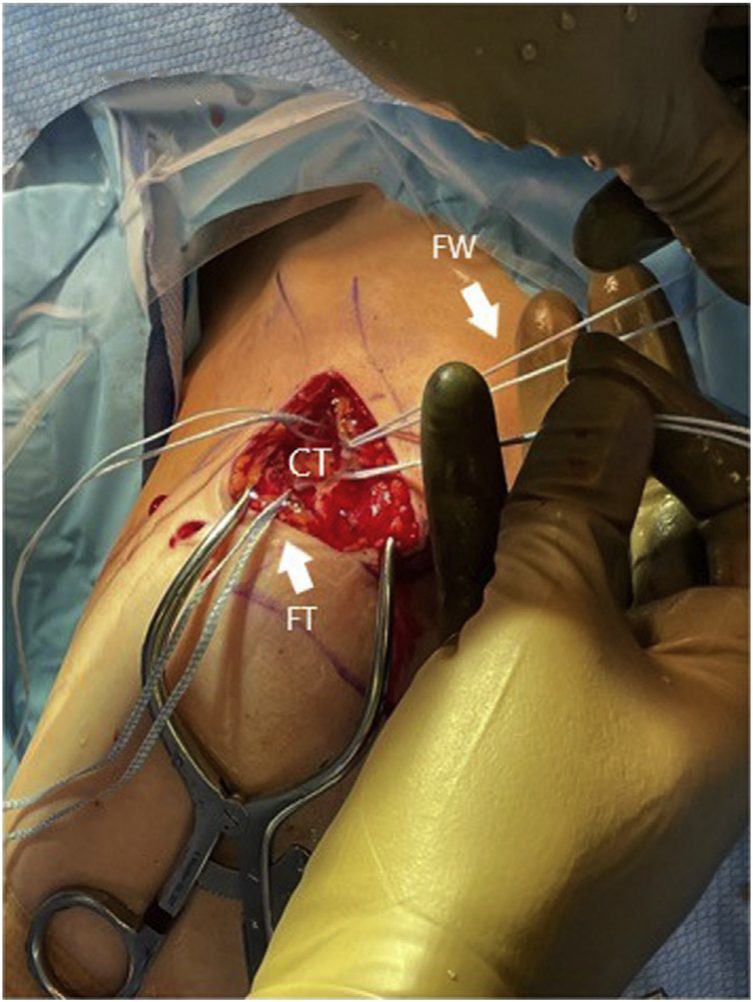
The same left shoulder is shown in the beach-chair position, looking from a lateral perspective. The FiberTape cerclage (FT) and the doubled-over #5 FiberWire (FW) are both pictured after passage around the coracoid (CT). The surgeon can be seen as he passes the tales of the FiberWire through its looped end to create a luggage tag around the coracoid.

**Fig 3:**
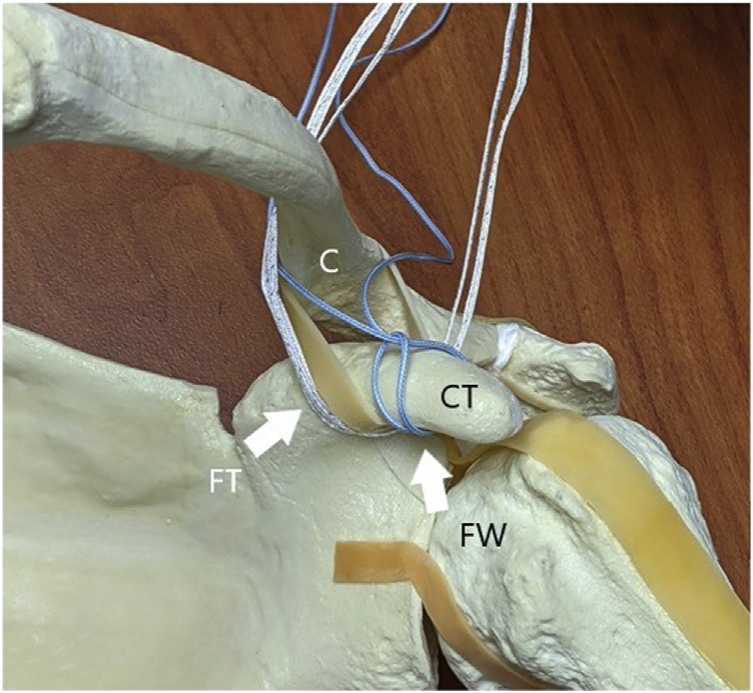
An anterior view of a sawbones model of a left shoulder is shown to demonstrate suture configuration. The #5 FiberWire (FW) has been used to create a luggage tag around the coracoid (CT). One of the FiberWire tails is then passed posterior to the clavicle (C) whereas the other tail is brought out anterior to the clavicle. The FiberTape cerclage (FT) is pictured after the free end of the cerclage has been passed from lateral to medial under the coracoid and then posteriorly beneath the clavicle.

Both of the tails from the #5 FiberWire are then passed through the looped end of the FiberWire to create a luggage tag configuration around the coracoid, as illustrated in Figure 2. A medium passing hook is then passed below the clavicle from posterior to anterior at the previously prepared location. One limb of the FiberWire luggage tag is brought through the nitinol shuttle loop along with the medial limb of the FiberTape Cerclage. The shuttle loop is then pulled to pass these sutures from anterior to posterior underneath the clavicle. A sawbones model of this configuration is shown in Figure 3.

### Step 5: Allograft Preparation and Passage

A fresh-frozen tibialis anterior allograft is prepared on the back table. Two #2 FiberLoop sutures on straight needles are used to perform a standard whip-stitch at both ends of the allograft. The passing hook is again inserted around the base of the coracoid from medial to lateral, and the sutures from one end of the allograft are shuttled around the base of the coracoid from lateral to medial using the same technique that was used for the FiberTape and FiberWire sutures. Visualization with the arthroscope can confirm that the allograft rests superficial to the FiberWire and cerclage sutures with respect to the coracoid as shown in the accompanying video. This is important to ensure that the sutures do not cut into the allograft. With the allograft then looped around the bottom of the coracoid, the medial sided allograft sutures are then used along with the passing hook to shuttle the medial end of the graft from anterior to posterior beneath the clavicle. At this point there should be 1 FiberWire tail, the free end of the FiberTape cerclage, and one end of the allograft exiting off the posterior edge of the clavicle. Exiting lateral to the coracoid and anterior to the clavicle should be the other FiberWire Tail, the lateral end of the FiberTape Cerclage (still configured on its white card), and the lateral end of the allograft.

### Step 6: Reduction of the Clavicle and Tensioning the Cerclage

**Fig 4:**
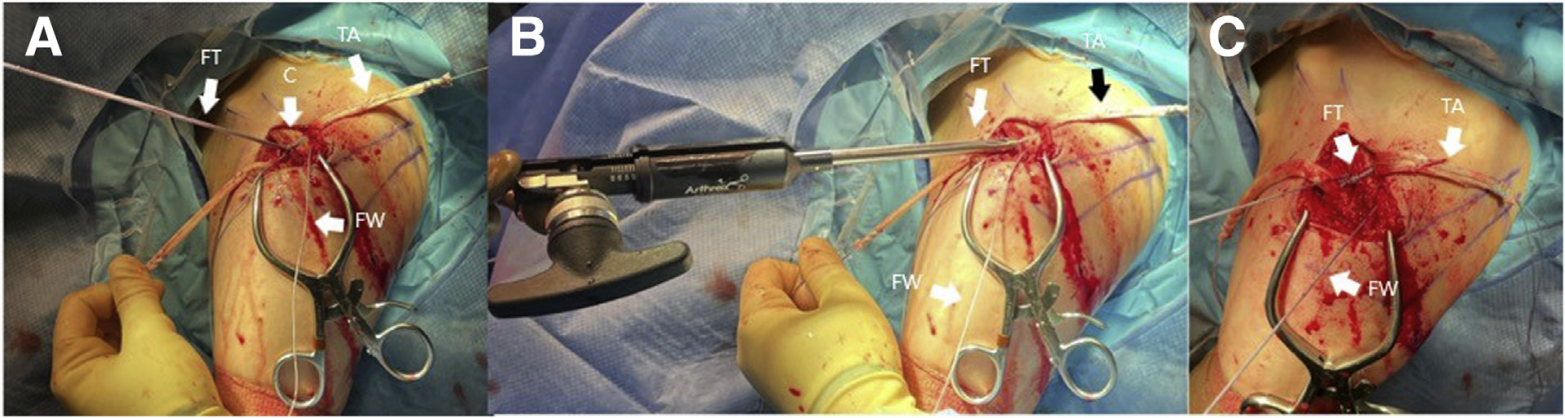
(A) The left shoulder is shown in the beach chair position, looking again from a lateral perspective. The tibialis anterior (TA) allograft is pictured after it has been passed beneath the coracoid and clavicle (C). The tail in the surgeon’s left hand is exiting lateral to the coracoid and anterior to the clavicle. The tail on the right is exiting medial to the coracoid and posterior to the clavicle. The FiberWire (FW) luggage tag is pictured after one tail has been shuttled posterior under the clavicle while the other remains anterior. The FiberTape cerclage (FT) is pictured after the free end has been shuttled through the pretied knot to create the cerclage construct. (B) The FiberTape cerclage (FT) is pictured loaded in the tensioning device in preparation for tensioning of the cerclage. A ball–spike pusher (not pictured) is used to achieve and hold reduction prior to tensioning. The tibialis anterior (TA) allograft and FiberWire (FW) remain in the same position. (C) The shoulder is shown after reduction of the clavicle and tensioning of the cerclage (FT). The cerclage sutures have been tied and cut. The tibialis anterior (TA) allograft and FiberWire (FW) remain in the same position.

The free tail of the cerclage suture is loaded through the shuttling loop on the lateral side of the cerclage (perform this as per packaging instructions on the white card in which the lateral end is loaded). The opposite end of shuttling loop is then pulled to draw the medial tail of the cerclage through a pretied knot in the FiberTape, see Figure 4A. A ball–spike pusher is then used to reduce the distal clavicle to the acromion. An assistant holds the reduction with the ball–spike pusher while the cerclage is tensioned using its accompanying tensioning device (Arthrex FiberTape cerclage tensioner with handle), as shown in Figure 4B. During this tensioning step, fluoroscopy can be used to ensure anatomic reduction of the acromioclavicular joint prior to proceeding forward with additional fixation. Once appropriate tensioning has been obtained, a knot is tied in the FiberTape tails after tensioning to prevent any possible slippage, as illustrated in Figure 4C.

### Step 7: Tying of the Luggage Tag Suture Tails and Allograft Tails Over the Clavicle

**Fig 5:**
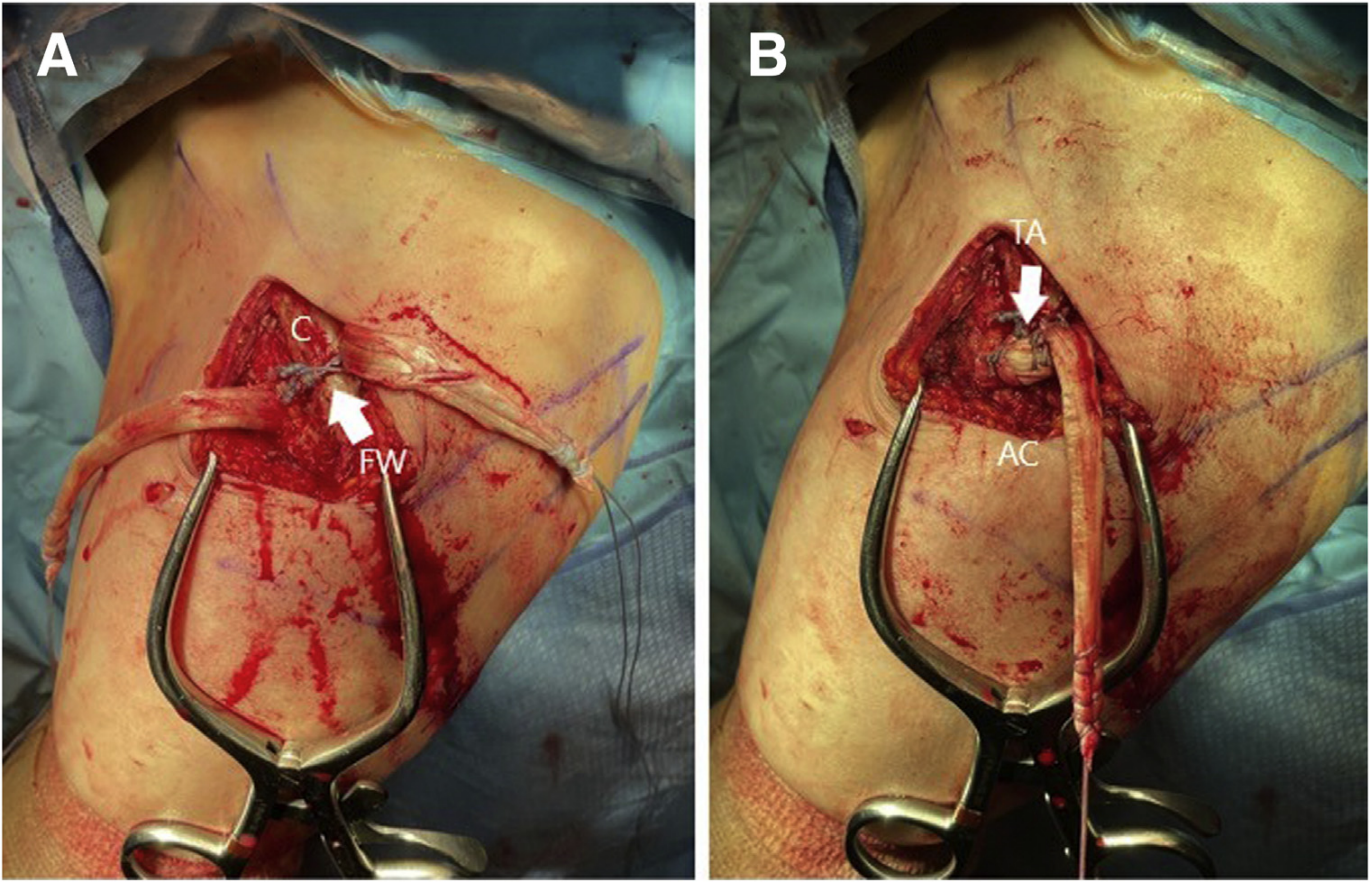
(A) The left shoulder is shown in the beach-chair position, looking from a lateral perspective. The tails of the FiberWire (FW) luggage tag have been tied over the clavicle (C) and cut. (B) The allograft (TA) has been tied over the top of the clavicle with the half-hitch knot sewn in place with #2 FiberWire sutures. The shorter limb of the graft has been amputated at the level of the knot while the longer limb is brought out laterally to the AC joint (AC).

With the cerclage holding the reduction, the 2 ends of the #5 FiberWire are tied tightly over the top of the clavicle. This suture augments the cerclage fixation and its short working length helps minimize the potential movement of the construct. The remaining tails of the cerclage and FiberWire are then cut so that only the knots remain atop the clavicle, as can be seen in Figure 5A. The allograft tails are then tied under tension over the top of the clavicle into a half-hitch configuration. One end of the allograft should be left long enough that it can be draped over to the AC joint after tying is complete, as shown in Figure 5B. A #2 FiberWire is then used, tying multiple knots through the allograft half-hitch to hold the graft in place and under tension in this configuration.

### Step 8: Augmentation of the AC Ligaments With the Remaining Tail of the Allograft

**Fig 6:**
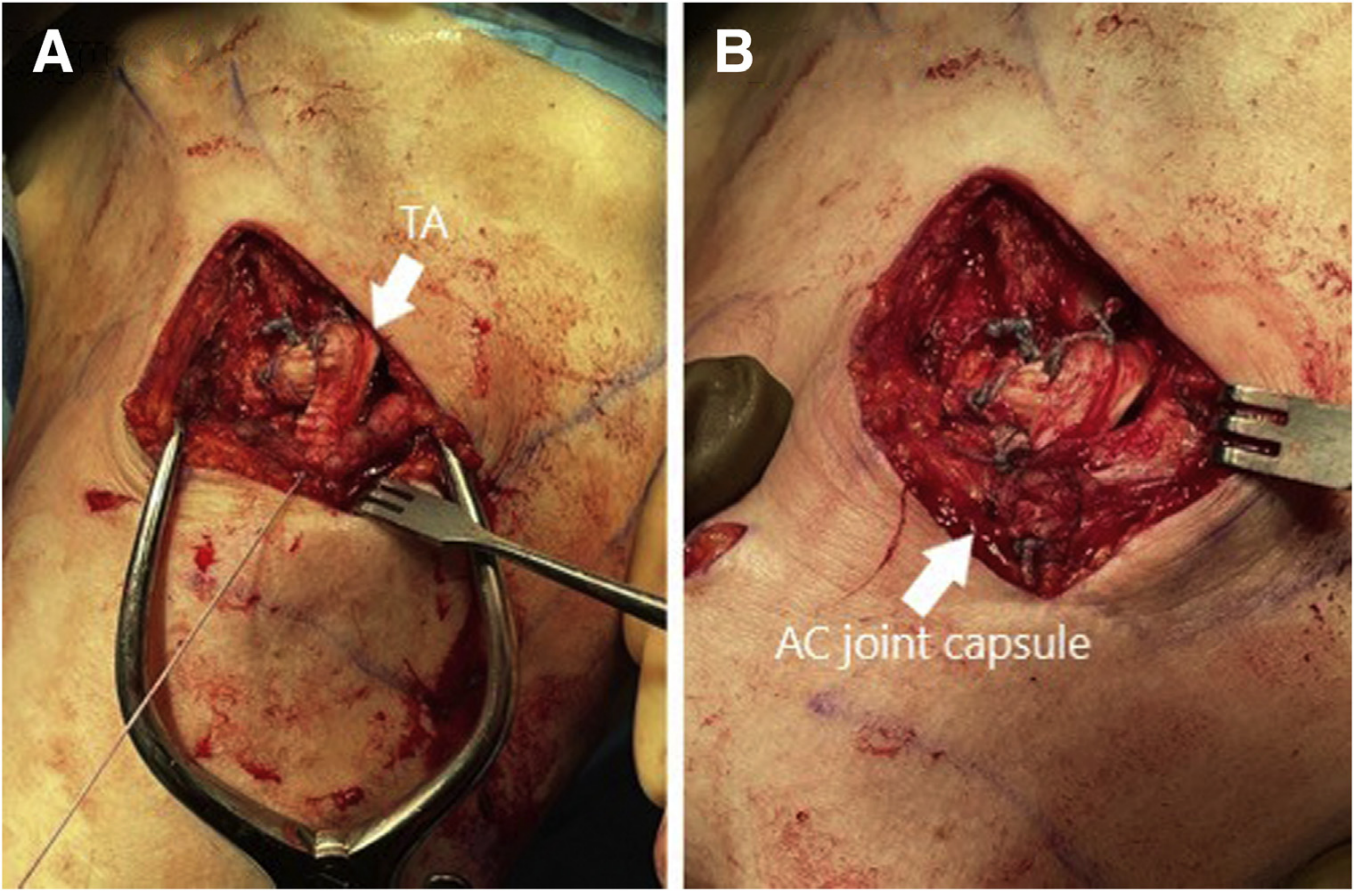
(A) The left shoulder is shown in the beach-chair position, looking from a lateral perspective. The long tail of the allograft has been cut at the level of the AC joint. The allograft (TA) has been placed beneath the lateral leaflet of the incised AC joint capsule. A #2 FiberWire is being used to tie the allograft to the posterior-superior aspect of the AC joint capsule. (B) The allograft has been sewn into the AC joint capsule with #2 FiberWire with the remaining AC joint capsule repaired around the allograft. The final coracoclavicular and AC joint reconstruction construct is pictured.

**Fig 7:**
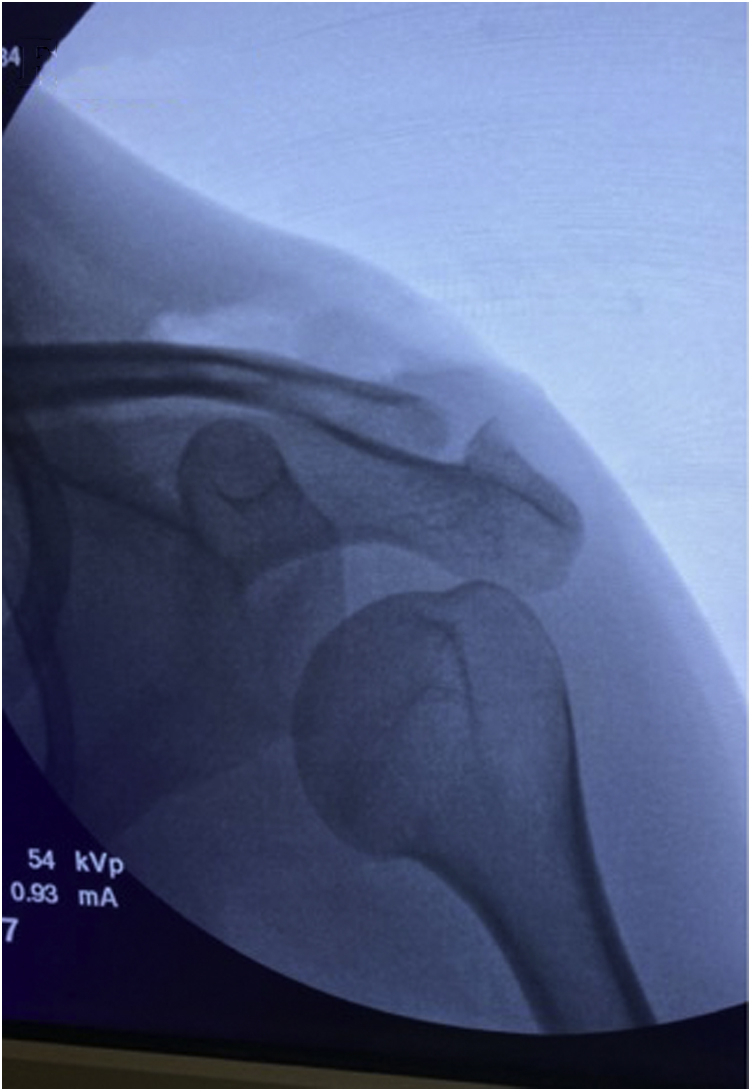
Intraoperative anteroposterior fluoroscopic view of left shoulder confirms reduction of the AC joint.

After the tied allograft has been appropriately sewn over the top of the clavicle, the shortest remaining end of the allograft can be amputated just above the half-hitch. The longer tail of the allograft is then draped over the AC joint and cut so that the remaining portion of the graft just reaches the acromial-sided flap of the previously sectioned AC ligaments as illustrated in Figure 6A. A #2 FiberWire suture is then used to sew the allograft tail to the acromial side of the posterior-superior AC joint capsule. The remaining AC joint capsule is then repaired together and to the allograft, as shown in Figure 6B. Final fluoroscopic views are obtained to confirm appropriate reduction of the AC joint as seen in Figure 7. The surgical wounds are closed according to surgeon preference. Overall advantages and disadvantages of this technique are shown in Table 3.

**Table 3:** Advantages and Disadvantages

Advantages	Disadvantages
Three forms of fixation should impart sufficient stability to maintain reduction even if one form of fixation fails	Requires surgeon to be comfortable and facile with arthroscopic techniques and suture management
Tunnel-free technique should eliminate bony failure through fracture	May have a steep learning curve if unfamiliar with methods of fixation
Hardware-free technique should obviate need for second surgery for implant removal	Limited long-term follow-up data
Can be used in both acute and chronic acromioclavicular joint injuries	

### Postoperative Protocol

The patient remains non–weight-bearing on the operative extremity in sling for a total of 6 weeks. Pendulums/Codman’s exercises are permitted immediately. Supine passive range of motion exercises with a 90° forward elevation and abduction limit are permitted beginning at 2 weeks’ postoperatively. Active assisted range of motion exercises, progressing to active range of motion is permitted beginning at 6 weeks’ postoperatively. Strengthening exercises may begin at 12 weeks’ postoperatively.

## Discussion

There are countless different described techniques to surgically treat acromioclavicular joint injuries, the majority of which have been investigated in the literature. Many systematic reviews have been performed and have shown comparable patient-reported outcomes with relatively low unplanned reoperation rates across all techniques. Overall complication rate was found to be up to 21.3% and overall reoperation rate was found to be up to 7.6%.^3^, ^4^, ^5^, ^6^, ^7^ Loss of reduction has been reported in up to 43% of cases, clavicle fracture in up to 18%, and infection in up to 7%.^3^, ^4^, ^5^, ^6^, ^7^ Use of hook plates and/or Kirshner wires were found to have the highest complication rate (26.3%) including plate loosening, acromial erosions and broken K-wires.^3^ Another recent review found a statistically significant re-operation rate when allograft was used.^9^

Many surgeons have sought to develop alternative techniques in effort to avoid many of these common complications. Several new variations have been described using a combination of suspensory fixation, allograft augmentation and/or lower profile implants.^10^, ^11^, ^12^, ^13^, ^14^ However, all of these recently published techniques still require at least one bony tunnel to be drilled in either the clavicle or the coracoid.

We have presented a technique that avoids drilling bone tunnels as well as uses multiple forms of fixation to achieve and maintain the reduction of the acromioclavicular joint. To our knowledge, this is the first reported technique that both avoids metal implants and does not necessitate drilling of bone tunnels. The absence of bone tunnels conveys the advantage of avoiding a stress riser in either the clavicle or coracoid. This confers a theoretical decrease in the risk of intra-operative or post-operative fracture of either bone. In addition, this is a largely implant-free technique which obviates the need for subsequent removal of hardware, both planned or unplanned. Perhaps most importantly, our technique uses multiple forms of fixation including both suspensory fixation with the FiberTape cerclage and the luggage tag FiberWire suture in conjunction with a tibialis anterior allograft. Should one form of fixation loosen or fail completely, there are two other forms of back-up fixation to maintain the reduction.

However, this technique is not without limitations. First, it requires that the surgeon be facile and comfortable with arthroscopic techniques to aid in passage of the suture and graft. Secondly, this technique does use a cadaveric allograft which does confer a risk of infection, potential adverse risk to foreign tissue or even increased risk of reoperation. Finally, this is a new technique with limited long-term follow up and no biomechanical analysis of stability. We acknowledge that additional clinical and biomechanical studies are required to directly compare this technique to previously described techniques. Nevertheless, our individual clinical and radiographic outcomes have so far been favorable.

## Supplementary Data

ICMJE author disclosure forms

Video 1 This video demonstrates the surgical technique for arthroscopically-assisted treatment of acromioclavicular (AC) joint instability in a way that avoids creation of bony tunnels through the clavicle or coracoid. In this case, a patient with a symptomatic complete high-grade AC separation undergoes surgical reconstruction.
